# ^13^C-Metabolic Flux Analysis Reveals the Metabolic Flux Redistribution for Enhanced Production of Poly-γ-Glutamic Acid in *dlt* Over-Expressed *Bacillus licheniformis*

**DOI:** 10.3389/fmicb.2019.00105

**Published:** 2019-02-01

**Authors:** Penghui He, Ni Wan, Dongbo Cai, Shiying Hu, Yaozhong Chen, Shunyi Li, Shouwen Chen

**Affiliations:** ^1^State Key Laboratory of Biocatalysis and Enzyme Engineering, Environmental Microbial Technology Center of Hubei Province, College of Life Sciences, Hubei University, Wuhan, China; ^2^Mechanical Engineering and Materials Science, Washington University, St. Louis, MO, United States; ^3^State Key Laboratory of Agricultural Microbiology, Huazhong Agricultural University, Wuhan, China

**Keywords:** *Bacillus licheniformis*, poly-γ-glutamic acid, ^13^C-metabolic flux analysis, *dltB*, cell surface negative charge

## Abstract

Poly-γ-glutamic acid (γ-PGA) is an anionic polymer with various applications. Teichoic acid (TA) is a special component of cell wall in gram-positive bacteria, and its D-alanylation modification can change the net negative charge of cell surface, autolysin activity and cationic binding efficiency, and might further affect metabolic production. In this research, four genes (*dltA, dltB, dltC*, and *dltD*) of *dlt* operon were, respectively, deleted and overexpressed in the γ-PGA producing strain *Bacillus licheniformis* WX-02. Our results implied that overexpression of these genes could all significantly increase γ-PGA synthetic capabilities, among these strains, the *dltB* overexpression strain WX-02/pHY-*dltB* owned the highest γ-PGA yield (2.54 g/L), which was 93.42% higher than that of the control strain WX-02/pHY300 (1.31 g/L). While, the gene deletion strains produced lower γ-PGA titers. Furthermore, ^13^C-Metabolic flux analysis was conducted to investigate the influence of *dltB* overexpression on metabolic flux redistribution during γ-PGA synthesis. The simulation data demonstrated that fluxes of pentose phosphate pathway and tricarboxylic acid cycle in WX-02/pHY-*dltB* were 36.41 and 19.18 mmol/g DCW/h, increased by 7.82 and 38.38% compared to WX-02/pHY300 (33.77 and 13.86 mmol/g DCW/h), respectively. The synthetic capabilities of ATP and NADPH were also increased slightly. Meanwhile, the fluxes of glycolytic and by-product synthetic pathways were all reduced in WX-02/pHY-*dltB*. All these above phenomenons were beneficial for γ-PGA synthesis. Collectively, this study clarified that overexpression of *dltB* strengthened the fluxes of PPP pathway, TCA cycle and energy metabolism for γ-PGA synthesis, and provided an effective strategy for enhanced production of γ-PGA.

## Introduction

Poly-γ-glutamic acid (γ-PGA) is an important anionic polypeptide consisting of D-glutamic acid and/or L-glutamic acid residues, which linked together via amide bonds between α-amino and γ-carboxyl (Feng et al., 2015; Sirisansaneeyakul et al., 2017). Since γ-PGA owns the excellent features of biocompatibility, biodegradability, water solubility, edibility, non-toxicity, environmentally friendliness, etc. (Cao et al., 2018), it has the wide-range applications in the areas of food, medicine, cosmetics industries, etc. (Cai et al., 2017).

*Bacillus* species have been proven as the efficient γ-PGA producers, and a number of metabolic engineering strategies have been developed to improve γ-PGA yield. For example, knocking out glutamate dehydrogenase genes *rocG* and *gudB* improved glutamic acid accumulation, which led to a 38% increase of γ-PGA yield in *Bacillus amyloliquefaciens* (Zhang et al., 2015). The synthesis of extracellular polysaccharide and lipopolysaccharide were blocked to decrease by-product yields for γ-PGA production in *B. amyloliquefaciens* LL3 (Feng et al., 2015). In addition, strengthening of NADPH and ATP supplies all benefited γ-PGA production (Cai et al., 2017, 2018). Recently, cell surface engineering was proven to be an effective strategy for enhancement production of metabolics. Overexpression of phosphatidylserine synthase gene *pssA* could enhance the cell membrane integrity and hydrophilicity, and further improved the cell tolerance and biorenewable yields (short-chain fatty acids, organic alcohols, organic acids and other aromatic compounds, etc) in *E. coli* (Tan et al., 2017). Elevation of membrane cardiolipin levels via overexpressing cardiolipin synthase gene *clsA* significantly increased hyaluronic acid titer by 204% in *Bacillus subtilis* (Westbrook et al., 2018). However, no research has been focused on the relationship between cell surface engineering and γ-PGA synthesis.

Teichoic acids (TAs) is an anionic polymer composed of ribitol and glycerol residues, and it was linked by phosphodiester bonds in the cell wall. The *dlt* operon, which consisted of four genes *dltA, dltB, dltC*, and *dltD*, owns the function of incorporating D-alanine to TAs in *Bacillus* (Koprivnjak et al., 2006). Previously, removal of D-alanyl esters from TAs of *Staphylococcus aureus* increased the amounts of Mg^2+^ bound to cell wall, which might affect the activities of several membrane proteins or enzymes (Lambert et al., 1975). Previous researches implied that the expression of *dlt* operon could regulate the cell surface net negative charge, and the changes on surface charge could mediate the recruitment of signaling molecules (lignin) (Hyyrylainen et al., 2000; Heit et al., 2011). γ-PGA synthesis is regulated by two component systems ComP∼ComA and DegS∼DegU, the regulators DegQ and SwrA (Tran et al., 2000). The recruitment of ComP might be affected by the reduction of cell surface negative charge, and further activated these regulators for γ-PGA synthesis. Furthermore, overexpression of *dltA* triggered the electrostatic repulsion between *S. aureus* and daptomycin, which represented the keystone of DAP resistance (Cafiso et al., 2014). γ-PGA and TAs are all anionic polymer, and the electrostatic repulsion generated between γ-PGA and TAs was not conducive to γ-PGA secretion. Thus, reducing cell surface negative charge might be an effective strategy for enhancement production of γ-PGA.

The biochemical reactions in cells were difficult to be profoundly described with the existing techniques. While, acting as a new method, ^13^C metabolic flux analysis (^13^C-MFA) is a promising tool to quantify cellular metabolism reaction rates in a network via isotopomer tracer experiments (Long et al., 2016). Moreover, ^13^C-MFA is a powerful tool to quantify the energy generation and consumption rates in the metabolic pathways (He et al., 2014; Yao et al., 2016), and it also applied to comprehend and analyze the changes in central carbon metabolism under aerobic and anaerobic conditions. For instance, the optimal tracers [1,2-^13^C] glucose, [1,6-^13^C] glucose, [1,2-^13^C] xylose, and [5-^13^C] xylose were applied in *Escherichia coli* under aerobic and anaerobic conditions, and their results demonstrated that the fluxes of EMP pathway and TCA cycle were all increased under anaerobic conditions, which generated more energy, formate, alcohol and succinate to adapt the adverse environments (Gonzalez et al., 2017).

In this study, the genes *dltA, dltB, dltC*, and *dltD* of *dlt* operon were, respectively, deleted and overexpressed in *B. licheniformis* WX-02, and ^13^C-MFA was performed to expound the effect of *dltB* overexpression on metabolic flux redistribution. In addition, the transcriptional level, by-products contents were also measured during γ-PGA synthesis. The aim of this study is to illustrate the relationship between *dlt* operon overexpression and γ-PGA synthesis by ^13^C-MFA, and provides an efficient strategy of strain improvement for γ-PGA production.

## Materials and Methods

### Strains and Culture Conditions

Strains and plasmids used in this study were provided in Table 1. *B. licheniformis* WX-02 was acted as the original strain for constructing recombinants, and *E. coli* DH5α was served as the host strain for plasmid construction. *B. licheniformis* and *E. coli* were grown in LB medium with responsible antibiotic (20 μg/mL kanamycin, 100 μg/mL ampicillin, or 20 μg/mL tetracycline), when required.

**Table 1:** The strains and plasmids used in this study.

Strains or plasmids	Description	Source of reference
***Bacillus licheniformis***		
WX-02	Poly-γ-glutamate producing strain (CCTCC M208065)	CCTCC
WX-02Δ*dltA*	WX-02 (Δ*dltA*)	This study
WX-02Δ*dltB*	WX-02 (Δ*dltB*)	This study
WX-02Δ*dltC*	WX-02 (Δ*dltC*)	This study
WX-02Δ*dltD*	WX-02 (Δ*dltD*)	This study
WX-02/pHY300	WX-02 harboring pHY300PLK	This study
WX-02/pHY-*dltA*	WX-02 harboring pHY-*dltA*	This study
WX-02/pHY-*dltB*	WX-02 harboring pHY-*dltB*	This study
WX-02/pHY-*dltC*	WX-02 harboring pHY-*dltC*	This study
WX-02/pHY-*dltD*	WX-02 harboring pHY-*dltD*	This study
**Plasmids**		
T_2_(2)-Ori	*Bacillus* knockout vector; Kan^r^	This study
T_2_-Δ*dltA*	T_2_(2)-Ori -*dltA* (A+B); to knock out *dltA*	This study
T_2_-Δ*dltB*	T_2_(2)-Ori -*dltB* (A+B); to knock out *dltB*	This study
T_2_-Δ*dltC*	T_2_(2)-Ori -*dltC* (A+B); to knock out *dltC*	This study
T_2_-Δ*dltD*	T_2_(2)-Ori -*dltD* (A+B); to knock out *dltD*	This study
pHY300PLK	*E. coli*-*B. licheniformis* shuttle vector, Ap^r^ (*E. coli*), Tc^r^ (*E. coli* and *B. licheniformis*)	This study
pHY-*dltA*	pHY300PLK containing P43 promoter, the gene *dltA* and *amyL* terminator	This study
pHY-*dltB*	pHY300PLK containing P43 promoter, the gene *dltB* and *amyL* terminator	This study
pHY-*dltC*	pHY300PLK containing P43 promoter, the gene *dltC* and *amyL* terminator	This study
pHY-*dltD*	pHY300PLK containing P43 promoter, the gene *dltD* and *amyL* terminator	This study

The γ-PGA fermentation medium contains 20 g/L glucose, 5 g/L NH_4_NO_3_, 11.35 g/L Na_2_HPO_4_, 8.15 g/L KH_2_PO_4_, 0.20 g/L MgSO_4_⋅7H_2_O, 0.01 g/L MnSO_4_⋅H_2_O, 0.0008 g/L CaCl_2_, and 0.0045 g/L Na_2_-EDTA. *B. licheniformis* were grown in twenty-four well plates with 2 mL working volume, and cultivated at 220 rpm and 37°C for 14 h. For ^13^C-MFA, glucose was replaced by [1, 2-^13^C] glucose (Sigma-Aldrich, CAS#138079-87-5) in γ-PGA production medium.

### Strain Construction

The construction procedures of *dltB* deletion and overexpression strains were served as the examples. For *dltB* deletion strain, the upstream and downstream arms of gene *dltB* were, respectively, amplified by the primers Δ*dltB*-F1/R1 and Δ*dltB*-F2/R2 (Supplementary Table S1), based on the genomic DNA of *B. licheniformis* WX-02, and fused by Splicing Overlapping Extension PCR (SOE-PCR) with primers Δ*dltB*-F1/R2. The fused fragment was inserted into the plasmid T_2_(2)-Ori at *Sac*I/*Xba*I, colony PCR and DNA sequence confirmed that the *dltB* deletion vector was constructed successfully, named as T_2_-Δ*dltB*. Then, T_2_-Δ*dltB* was electro-transferred into *B. licheniformis* WX-02, and the positive transformant was cultivated in LB medium with 20 μg/mL kanamycin at 220 rpm and 45°C, and sub-cultured for three times to obtain the single-crossover recombinants. The recombinants were grown in LB medium at 37°C with six subcultures, and the kanamycin sensitive colonies were further confirmed by colony PCR and DNA sequence, and the *dltB* deletion strain was named WX-02Δ*dltB*.

For gene overexpression strain, P43 promoter from *B. subtilis* 168, gene *dltB* and *amyL* terminator from *B. licheniformis* WX-02 were, respectively, amplified (Supplementary Table S1), and fused by SOE-PCR. The fused fragment was inserted into pHY300PLK at the restriction sites *Eco*RI/*Xba*I, resulting in the plasmid pHY-*dltB*. Then, pHY-*dltB* was introduced into WX-02 by electro-transformation, resulting in the *dltB* overexpression strain, named WX-02/pHY-*dltB*.

### Analytic Methods

The cell biomass was monitored by measuring OD_600_ using a UV-spectrophotometer-752 N (Shanghai Instrument Analysis Instrument Co., Ltd., Shanghai, China), glucose concentrations were determined via using the Enzyme Electrode Analyzer SBA-40E (Shandong Academy of Sciences, Shandong, China). The γ-PGA yield was measured according to our previously reported method (Cai et al., 2018). Briefly, the volume of 2 mL fermentation broth was mixed with 4 mL distilled water, and cells were separated by centrifugation at 12000 rpm for 6 min after adjusting the pH to 2.5∼3.0. While, three volumes of absolute ethanol were added into the supernatant after adjusting pH to 7.0, and the precipitate was dried at 80°C to a constant weight for measuring γ-PGA yield. The net negative charge was measured by determining the cation binding rate of cell surface in LB medium, according to the previously described method (Cao et al., 2017; Chen et al., 2018).

The concentrations of by-products, acetoin, 2,3-butanediol and acetic acid, were measured by GC/MS (Gas chromatograph Trace, Thermo; Triple Quadrupole Mass Spectrometer, Thermo; column: DB-WAXMS, 30 m × 0.25 mm × 0.25 μm, Thermo; United States). Briefly, the volume of 0.5 mL fermentation broth was mixed with 1.5 mL absolute ethanol, and the supernatant was separated by centrifugation at 12000 rpm for 10 min. Equal volume of ethyl acetate was added into the supernatant for extracting acetoin, 2,3-butanediol and acetate. The parameters of GC-MS were set as follow: Gas chromatographic inlet temperature was 220°C, the injection volume was 1 μL, carrier gas flow rate was 1 mL/min and the split ratio was 20:1. The temperature program: hold at 40°C for 1 min, and increased to 160°C at 6°C/min, hold for 1.5 min, and then raised to 220°C at 30°C/min, hold for 2 min. Solvent delay was set as 4 min. The range of mass to charge ratio (m/z) in MS was set between 35 and 500.

The gene transcriptional level were measured with TRIzol^®^ Reagent (Invitrogen, United States) and PrimeScript^TM^ II 1st Strand cDNA Synthesis Kit (TaKaRa, Japan) according to our previously reported method (Cai et al., 2017). The primers used for RT-qPCR were listed in Supplementary Table S2, and the housekeeping gene 16S rRNA was used to normalize the gene expression data. The data were averaged and presented as the mean ± SD.

### Mass Isotopomer Distribution Analysis of Amino Acids

To analyze the mass isotopomer distributions (MIDs) of amino acids, [1,2-^13^C] glucose was served as the sole carbon source for cell growth and γ-PGA synthesis. The cell pellets at the mid-logarithmic phase (7 h) were collected and hydrolyzed by 6 M HCl at 100°C for 24 h. The supernatant of hydrolyzate was air-dried, and the precipitate was subsequently derivatized with *N*-tert-butyldimethylsilyl-N-methyltrifluoroacetamide (TBDMS). Then, the derivatization products were quantified by GC/MS (Gas chromatograph Trace, Thermo; Triple Quadrupole Mass Spectrometer, Thermo; column: TG-5MS, 30 m × 0.25 mm × 0.25 μm, Thermo; United States). The injection volume and injection split ratio were 1 μL and 1:10, respectively, and carrier gas helium was 1.2 mL/min. GC temperature program was set as follows: hold at 150°C for 2 min, and increased to 280°C at 3°C/min, and increased to 300°C at 20°C/min, and then hold for 5 min. Solvent delay was set as 5 min, and the range of mass to charge ratio (m/z) in MS was set between 60 and 500 (You et al., 2012). The software WuFlux-Ms Tool was used to analyze and correct amino acid MS data (fragments of [M-57]^+^, [M-159]^+^, or [M-85]^+^, and [f302]) (Wahl et al., 2004). Isotopomer labeling fractions (M0, M1, M2, etc.) represent fragments containing unlabeled, singly labeled, doubly labeled amino acids, etc (Wan et al., 2017).

### ^13^C-Metabolic Flux Analysis With INCA

^13^C-MFA was performed by using the INCA software (Young, 2014), which is based on the elementary metabolite units (EMU) framework (Antoniewicz et al., 2007). The central metabolic network was modified according to the previous research (He et al., 2017) and KEGG pathway database. As the target product and main by-products, several steps for γ-PGA, acetoin and 2,3-butanediol syntheses, such as 2 Pyruvate → α-acetolactate + CO_2_, α-acetolactate → Acetoin + CO_2_, Acetoin+ NADH ↔ 2,3-Butanediol + NAD^+^, Acetoin+ NAD^+^ → Acetyl-CoA + NADH, Acetoin + ATP → Acetoin_ex, 2,3-Butanediol → 2,3-Butanediol_ex, Glutamate + ATP → γ-PGA, and γ-PGA → γ-PGA_ex, were added into the metabolic network (seeing in the Supplementary Table S4). The information of labeling amino acids (alanine, glycine, valine, leucine, serine, theronine, phenylalanine, aspartic acid, glutamic acid, and histidine) (Supplementary Table S3) were substituted into the model for calculating metabolic flux of central metabolism (Wan et al., 2017). The standard deviation of ^13^C-enrichment was set as 0.0107 for statistical analysis (Nanchen et al., 2007), and the flux estimations were performed by using MATLAB R2014a (The Mathworks Inc.).

### Statistical Analyses

All samples were analyzed in triplicate, and the data were presented as the mean ± SD for each sample point. Significant differences were determined by one-way analysis of variance (ANOVA). Statistical significance was defined as *P* < 0.05.

## Results

### Effects of Deletion and Overexpression of *dlt* Operon on γ-PGA Production

**FIGURE 1:**
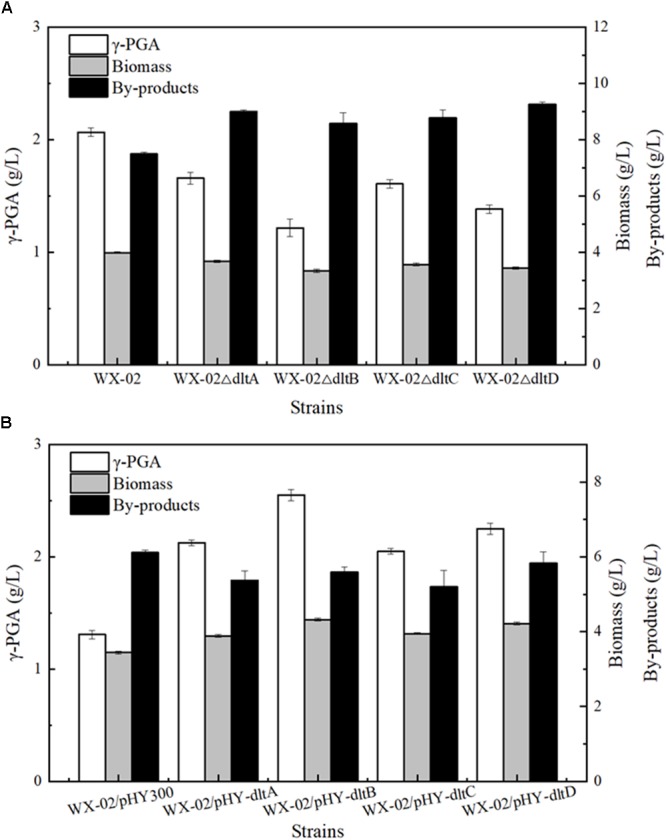
Effects of deletion and overexpression of *dlt* operon on γ-PGA and by-products production. **(A)** The γ-PGA yields, biomass formation and by-products yields of WX-02, WX-02Δ*dltA*, WX-02Δ*dltB*, WX-02Δ*dltC*, and WX-02Δ*dltD*. **(B)** The γ-PGA yields, biomass formation and by-products yields of WX-02/pHY300, WX-02/pHY- *dltA*, WX-02/pHY-*dltB*, WX-02/pHY-*dltC*, and WX-02/pHY-*dltD*.

To evaluate the relationship between *dlt* operon expression and γ-PGA synthesis, the genes *dltA, dltB, dltC* and *dltD* of *dlt* operon were deleted and overexpressed in the γ-PGA production strain *B. licheniformis* WX-02, resulting in the recombinant strains WX-02Δ*dltA*, WX-02Δ*dltB*, WX-02Δ*dltC*, WX-02Δ*dltD* and WX-02/pHY-*dltA*, WX-02/pHY-*dltB*, WX-02/pHY-*dltC*, WX-02/pHY-*dltD*, respectively. Based on our results of Figure 1, deletions of *dltA, dltB, dltC*, and *dltD* all reduced γ-PGA yield, which decreased by 19.81, 41.55, 22.22, and 33.33% compared with that of control strain WX-02 (2.07 g/L), respectively (Figure 1A). And deletion of one gene has no effects on the transcriptional levels of other genes involved in *dlt* operon (Supplementary Figure S2). Furthermore, γ-PGA yields produced by the *dlt* overexpression strains were 2.13, 2.55, 2.05, and 2.25 g/L, increased by 62.21, 94.66, 56.49, and 71.76% compared to WX-02/pHY300 (1.31 g/L), respectively (Figure 1B). Collectively, these above results indicated that overexpression of *dlt* operon benefited γ-PGA synthesis.

**FIGURE 2:**
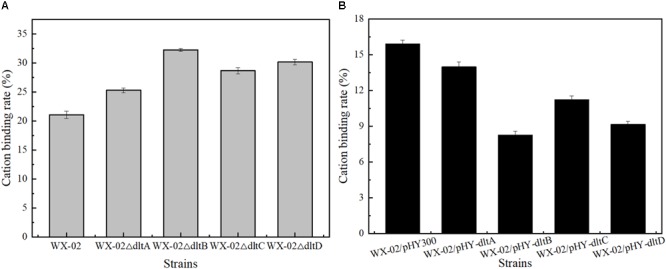
Effects of deletion and overexpression of *dlt* operon on the cation binding rate. **(A)** The cation binding rates of WX-02, WX-02Δ*dltA*, WX-02Δ*dltB*, WX-02Δ*dltC*, and WX-02Δ*dltD*. **(B)** The cation binding rates of WX-02/pHY300, WX-02/pHY-*dltA*, WX-02/pHY-*dltB*, WX-02/pHY-*dltC*, and WX-02/pHY-*dltD*.

Previously, the *dlt* operon was proved to play the important role on the net negative charge of cell surface. As shown in Figure 2, the negative charges of cell surface were all increased in the *dlt* deletion strains, and decreased in the *dlt* overexpression strains. The negative charge was reduced by 48.93% in the *dltB* overexpression strain WX-02/pHY-*dltB*.

### Fermentation Process Curves of WX-02/pHY300 and WX-02/pHY-*dltB*

**FIGURE 3:**
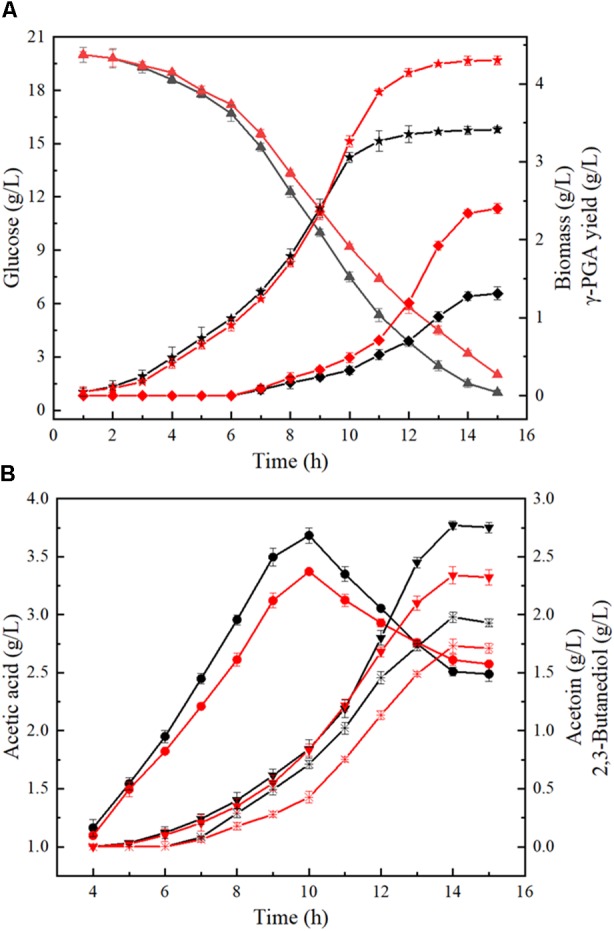
Fermentation process curves of WX-02/pHY300 and WX-02/pHY-*dltB*. **(A)** Biomass, glucose concentration, and γ-PGA yield. **(B)** Acetic acid, acetion, and 2,3-butanediol yields. The solid black line indicates the control strain WX-02/pHY300, and the red solid line represents the recombinant strain WX-02/pHY-*dltB*. Triangle, glucose; pentagram, biomass; diamond, γ-PGA; circle, acetic acid; inverted triangle, 2,3-butanediol; fork, acetoin.

Furthermore, the process curves of WX-02/pHY-300 and WX-02/pHY-*dltB* were determined, and the cell growth, glucose uptake, γ-PGA yield, by-product (acetoin, 2,3-butanediol and acetic acid) concentrations were measured during γ-PGA production. As shown in Figure 3A, the cell biomass of WX-02/pHY-*dltB* were slightly lower than those of control strain WX-02/pHY-300 before 9 h, and then increased faster subsequently. Besides, the logarithmic growth period is delayed by 2 or 3 h, and there is no obvious decline phase in the *dltB* overexpression strain WX-02/pHY-*dltB*. The specific cell growth rate of WX-02/pHY-*dltB* was 0.32 h^-1^, increased by 7.58% compared with that of WX-02/pHY-300 (Table 2). The glucose uptake rate of WX-02/pHY-*dltB* was 7.32 mmol/g DCW/h, similar with that of WX-02/pHY-300 (7.23 mmol/g DCW/h), and the γ-PGA synthetic rate of WX-02/pHY-*dltB* (0.92 mmol/g DCW/h) was increased by 41.54% compared to WX-02/pHY-300 (0.65 mmol/g DCW/h). The concentrations of by-products (acetoin, 2,3-butanediol and acetic acid) was significantly lower in WX-02/pHY-*dltB* than that of WX-02/pHY-300 (Figure 3B). Moreover, the formation rates of acetic acid and 2,3-butanediol showed no significant difference between these strains, while the acetoin formation rate was decreased by 50.00% in WX-02/pHY-*dltB* (0.56 mmol/g DCW/h) (Table 2). Assuming that there was no other significant by-products and CO_2_ produced, and the carbon recoveries of WX-02/pHY300 and WX-02/pHY-*dltB* were roughly consistent for each strain (Table 2).

**Table 2:** The fermentation characteristics of WX-02/pHY300 and WX-02/pHY-*dltB*.

Strains	Specific growth rate μ (h^-1^)	Glucose uptake rate (mmol/g/h)	γ-PGA formation rate (mmol/g/h)	Acetate formation rate (mmol/g/h)	Acetoin formation rate (mmol/g/h)	2,3-Butanediol formation rate (mmol/g/h)	carbon recovery (mmol/g/h)
WX-02/pHY300	0.29	7.23	0.65	3.48	1.12	1.14	31.04
WX-02/pHY-*dltB*	0.32	7.32	0.92	3.57	0.56	1.10	31.39

*The carbon recoveries of WX-02/pHY300 and WX-02/pHY-*dltB* were calculated as follows:*

*In control strain WX-02/pHY300:*

*Carbon consumption of glucose: 7.23^∗^6 = 43.38 mM/g/h.*

*Carbon consumption for biomass and product synthesis:*

*0.65^∗^5+3.48^∗^2+1.12^∗^4+1.14^∗^4+0.29/24.6^∗^1000 = 31.04 mM/g/h.*

*In engineered strain WX-02/pHY-*dltB*:*

*Carbon consumption of glucose: 7.32^∗^6 = 43.92 mM/g/h.*

*Carbon consumption for biomass and product synthesis:*

*0.92^∗^5+3.57^∗^2+0.56^∗^4+1.10^∗^4+0.32/24.6^∗^1000 = 31.39 mM/g/h.*

*(Biomass was calculated by C_*1*_H_*1.8*_O_*0.5*_N_*0.2*_. The calculation of carbon recovery is products formation rate ^∗^ the number of carbon).*

### ^13^C-Metabolic Flux Analysis

The metabolic flux distributions were determined based on the measured mass isotopomer distributions of proteinogenic amino acids and synthetic rates of γ-PGA, acetic acid, acetoin and 2,3-butanediol. The measured mass isotopomer distributions (MIDs) of proteinogenic amino acids coincided well with the simulated MIDs (Supplementary Table S3), indicating the good fit and high flux precision between the measured and simulated data (Yao et al., 2016). The measured biomass formation rates were not employed as the constraints of ^13^C-MFA model (Table 2). The flux values and exchange coefficients were estimated with the ^13^C MFA model listed in Supplementary Table S4.

**FIGURE 4:**
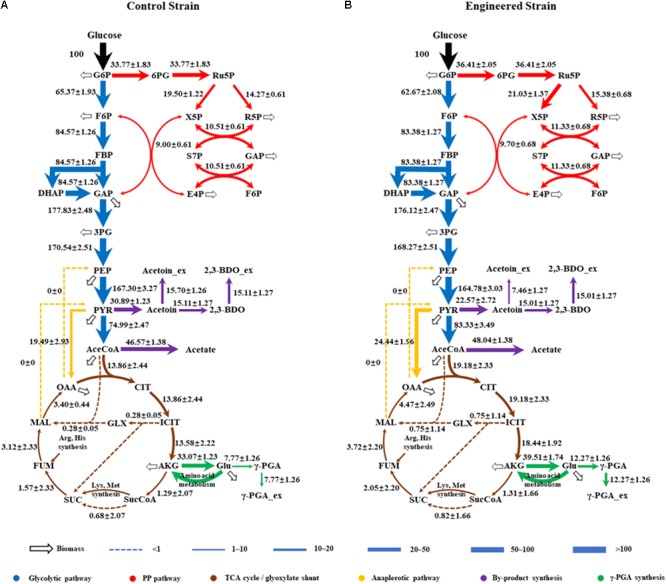
Metabolic flux distributions in the central metabolic pathways of WX-02/pHY300 and WX-02/pHY*-dltB* during exponential growth on [1,2-^13^C]glucose. Fluxes shown are normalized to glucose uptake rate of 100 for each strain (estimated flux ± SD). **(A)** WX-02/pHY300. **(B)** WX-02/pHY-*dltB*.

As shown in Figure 4, the fluxes in the central metabolism of *B. licheniformis* were redistributed in WX-02/pHY-*dltB*. Firstly, the flux from pyruvate to acetyl-CoA was increased by 11.12%, and the flux distributed into tricarboxylic acid cycle was enhanced by 38.38% in WX-02/pHY-*dltB*. The flux for acetic acid production was increased slightly, whereas, the flux of overflow metabolism (from pyruvate to acetoin and 2,3-butanediol) was decreased by 26.93%. Secondly, the flux of PP pathway was increased by 7.82% in the *dltB* overexpression strain, which might generate more NADPH for γ-PGA synthesis. Thirdly, the flux through pyruvate carboxylation (PYR + CO_2_ → OAA), the major anaplerotic flux into TCA cycle, was increased by 25.40% in WX-02/pHY-*dltB*. The increase of pyruvate carboxylation could enable more carbon flow from glucose to TCA cycle, and further increased the demand for oxaloacetate based biomass synthesis (Table 2; He et al., 2014). Fourthly, the fluxes from α-ketoglutaric acid to glutamic acid and γ-PGA were increased by 19.47 and 57.92%, respectively. Fifthly, the flux of EMP pathway was decreased by 4.13%, which further reduced the NADH generation and by-products (acetoin, 2,3-butanediol and acetic acid) syntheses. Lastly, the estimated biomass biosynthesis flux was slightly higher (about 6.40%) in WX-02/pHY-*dltB* (Figure 4), which positively correlated with the results of cell biomass (Table 1).

The cofactors (such as NADH, NADPH, etc.) and ATP play the important roles in metabolic production (Kind et al., 2013), as well as for γ-PGA synthesis (Cai et al., 2017, 2018). Based on our metabolic flux model in *B. licheniformis*, the NADPH and ATP formation rates in WX-02/pHY-*dltB* were increased by 12.50 and 3.54%, which were beneficial for γ-PGA synthesis (Table 3).

**Table 3:** The synthesis and consumption contents of NADPH, NADH, ATP, and FADH2 of WX-02/pHY300 and WX-02/pHY-*dltB* (mmol/g CDW/h).

Pathway	NADPH		NADH		FADH2		ATP	
	Control	Engineered	Control	Engineered	Control	Engineered	Control	Engineered
Glycolysis	0	0	177.83	176.12	0	0	160.56	157.52
PP pathway	67.54	72.82	0	0	0	0	0	0
TCA cycle	13.58	18.44	79.68	89.11	1.57	2.05	0.68	0.82
Amino acid synthesis	–58.67	–67.08	3.79	4.08	0	0	–23.36	–25.11
γ-PGA formation	0	0	0	0	0	0	–7.77	–12.27
Acetic acid formation	0	0	0	0	0	0	46.57	48.04
Acetoin and 2,3-butanediol formation	0	0	–15.02	–14.91	0	0	–15.70	–7.46
Biomass formation	–22.47	–24.19	6.10	6.56	0	0	–139.32	–149.96
Oxidative phosphorylation	0	0	–253.93	–262.64	–1.57	–2.05	764.93	792.02

*All the flux values are normalized to the glucose uptake rate of 100 mmol h^-1^ for each strain.*

### Transcriptional Level Analysis

**FIGURE 5:**
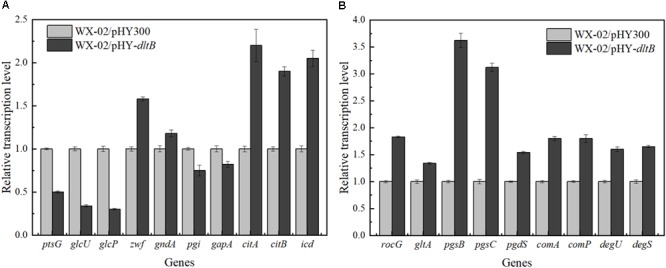
Transcription levels of genes in glucose metabolism and γ-PGA synthesis in WX-02/pHY300 and WX-02/pHY-*dltB*. **(A)** Genes in glucose metabolism. **(B)** Genes in γ-PGA synthesis.

The transcriptional levels of genes related to glucose metabolism and γ-PGA synthesis were measured in WX-02/pHY300 and WX-02/pHY-*dltB* (Figure 5). Compared to WX-02/pHY300, the transcription levels of glucose transporter genes *ptsG, glcU*, and *glcP* were reduced obviously in *dltB* overexpression strain. The glucose-6-phosphate dehydrogenase gene *zwf* in PP pathway showed higher expression levels, while the transcription level of glucose-6-phosphate isomerase gene *pgi* in EMP pathway was decreased by 25.01%, indicating the elevated PP pathway and depressed EMP pathway fluxes in WX-02/pHY-*dltB*. In addition, the transcription levels of genes *citB* (encoding citrate synthase) and *icd* (encoding isocitrate dehydrogenase) in TCA cycle were enhanced by 90.03 and 105.12%, which strengthened the metabolic flux in TCA cycle.

Furthermore, the transcription levels of glutamate dehydrogenase gene *rocG*, which mainly catalyzes the formation of glutamic acid from α-ketoglutaric acid in *B. licheniformis* WX-02 (Tian et al., 2017), was improved by 83.25% in WX-02/pHY-*dltB*, and γ-PGA synthetase genes *pgsB, pgsC* were, respectively, increased by 2.62- and 2.12-fold, which indicated that more glutamic acid was synthesized and converted to γ-PGA in WX-02/pHY-*dltB*. Besides, the gene *pgdS* (encoding γ-DL-glutamyl hydrolase) was also increased in WX-02/pHY-*dltB*, and the improvement of *pgdS* could decrease the molecular weight of γ-PGA (Tian et al., 2017), positively correlated with the actual measurement results (Date not shown). Moreover, transcription factor genes *comA, comP, degS*, and *degU*, which associated with the synthesis of γ-PGA, were all increased in the *dltB* overexpression strain.

## Discussion

Cell wall and cell membrane are the selective permeation barrier of bacteria, and engineering of cell surface negative charge, membrane phospholipid head and fatty acid hydrophobic tail composition could affect bacterial growth and product production (Hyyrylainen et al., 2007; Ghorbal et al., 2013; Tan et al., 2017). Previous researches demonstrated that the increases of negative charge on the cell surface could improve the secretion efficiency of target protein, and the increase rate of target protein with lower isoelectric points (PI) was higher than that of protein with higher PI (Cao et al., 2017; Chen et al., 2018). While, based on our results, the increases of cell surface negative charge led to the decrease of γ-PGA yield in the *dlt* operon mutants.

On the one hand, overexpression of *dltB* slowed down the absorption and utilization rate of glucose (Figure 3A), and further decreased the flux of EMP pathway. Previous research implied that reduction of glycolysis flux in *E. coli* THRD could reduce the accumulation of acetate and increase NADPH and L-threonine generations (Xie et al., 2014), which was consistent with our results (Figure 3A and Table 2). Moreover, the alanylation of teichoic acids could modulate the negative charge of cell wall to protect secretory or cell wall-associated proteins against degradation during the post-translocational folding (Hyyrylainen et al., 2000), and enhancement of cell surface negative charge could increase the binding capacity of autolysin, and accelerated autolysis of bacteria (Steen et al., 2005). Figure 3A showed that the logarithmic period of WX-02/pHY-*dltB* was extended by 2∼3 h, and the growing status of WX-02Δ*dltB* was obviously badness in post-fermentation compared with WX-02 (Supplementary Figure S1). This results indicated that overexpression of *dltB* could promote the cell growth by reducing the autolysis of cells. Therefore, overexpression of *dltB* reduced the absorption and utilization rate of glucose, which decreased the flux of EMP pathway and synthesis capabilities of by-products (acetoin, 2,3-butanediol and acetic acid), and extended logarithmic growth phase, and all these phenomenons were beneficial for γ-PGA synthesis.

Then, ^13^C-labeled isotope tracer and ^13^C-MFA were applied to further evaluate the metabolic flux redistributions in the *dltB* overexpression strain. Based on our results, the flux of EMP pathway is weakened, which reduced the NADH generation and by-products synthesis, and our results were positively consisted with the previous research (Lee and Oh, 2016). Secondly, the flux of PPP pathway and NADPH supply were all enhanced in the *dltB* overexpression strain. Since the conversion of α-ketoglutarate to glutamic acid requires NADPH in *B. licheniformis* WX-02, enhancement of NADPH supply contributes to γ-PGA synthesis (Cai et al., 2017; Tian et al., 2017). In addition, the flux of TCA cycle was enhanced by 38.38% in the *dltB* overexpression strain, and the increase of α-ketoglutaric acid synthesis flux was beneficial for cell growth (Wan et al., 2017) and γ-PGA production. Besides, the flux of complement-deficient pathway (PYR → OAA) was enhanced by 25.40%, which is beneficial for oxaloacetate accumulation and cell growth (Sauer et al., 1999). Finally, the flux from α-ketoglutarate to glutamate and further generate γ-PGA were, respectively, increased by 19.47 and 57.92%, which were consistent with fermentation characters (Figure 1).

Furthermore, overexpression of *dlt* operon led to the decrease of cell surface negative charge (Figure 2), and further changed the extracellular microenvironment (Hyyrylainen et al., 2000), which might promote the recruitment of signal molecular ComP to activate two component system ComP-ComA for γ-PGA synthesis. Based on our results, the transcriptional levels of *comP*/*comA* and *degU*/*degS* were all increased in the *dltB* overexpression strian, which further affected the transcription levels of genes involved in γ-PGA synthesis and carbon metabolism (Figure 5). Moreover, the decrease of cell surface negative charge could reduce the binding of cationic (e.g., Mg^2+^ and Ca^2+^, etc.) and cationic antimicrobial peptides on cell surface (Neuhaus and Baddiley, 2003), and the low levels of cationic or cationic antimicrobial peptides could activate PhoQ(R)∼PhoP system (Garcia Vescovi et al., 1996; Ren et al., 2017). In this study, the concentration of phosphate in the culture medium was too high to activate PhoQ(R)∼PhoP system (Devine, 2018) in WX-02/pHY300, however, due to the low level of Mg^2+^ and Ca^2+^ on cell surface of *dltB* overexpression strain, the PhoQ(R)∼PhoP might be activated in WX-02/pHY-*dltB*, which was consistent with our unpublished results that strengthening PhoQ(R)∼PhoP system benefits γ-PGA synthesis. Finally, γ-PGA is an anionic polypeptide, and the decrease of cell surface negative charge might reduce the strength of electrostatic repulsion (Cafiso et al., 2014) between TAs and γ-PGA, which further benefited γ-PGA secretion. To test this hypothesis, the negatively charged products (such as lichenysin, bacitracin, pulcherrimin, et al.) were analyzed, and our results showed that the yields of those products with negative charge were all increased in *dlt* overexpression strains (Supplementary Figure S3).

## Conclusion

Cell surface engineering is a promising tactic for enhanced production of metabolites. This study demonstrated that overexpression of *dlt* operon could increase γ-PGA production for the first time. Furthermore, ^13^C-MFA was applied to illustrate the affect of *dltB* overexpression on γ-PGA synthesis. Our results showed that overexpression of *dltB* could reduce the negative charge of cell surface, which further reduced the absorption and utilization rate of glucose and the flux of EMP pathway. Meanwhile, the increases of TCA cycle flux, NADPH and ATP supplies, glutamic acid formation benefited γ-PGA synthesis. This work demonstrated that ^13^C-MFA provided the distinct metabolic insights in engineered microbes for the changes in central carbon metabolism.

## Author Contributions

PH and SC designed the study. PH, SH, and YC carried out the molecular biology studies and construction of recombinant strains. PH, DC, SH, and YC carried out the fermentation studies. PH and NW carried out the metabolic flux analysis. PH, DC, SL, and SC analyzed the data and wrote the manuscript. All authors read and approved the final manuscript.

## Conflict of Interest Statement

The authors declare that the research was conducted in the absence of any commercial or financial relationships that could be construed as a potential conflict of interest.

## Supplementary Material

All the primers sequences for strain construction and RT-qPCR were listed in Supplementary Tables S1 and S2, respectively. The results of labeled amino acids were provided in Supplementary Table S3, and the specific data in metabolic pathway were provided in Supplementary Table S4.

## Abbreviations

2,3-BDO: 2,3-butanediol
3PG: 3-phosphoglyceric acid
6PG: 6-phosphogluconolactone
AceCoA: acetyl-CoA
AKG: α-ketoglutarate
CIT: citrate
DHAP: dihydroxyacetone phosphate
E4P: erythrose 4-phosphate
EMP pathway: glycolysis pathway
F6P: fructose 6-phosphate
FBP: fructose 1,6-bisphosphate
FUM: fumarate
G6P: glucose-6-phosphate
GAP: glyceraldehyde 3-phosphate
Glu: glutamic acid
GLX: glyoxylate
ICIT: isocitrate
MAL: malate
OAA: oxaloacetate
PEP: phosphoenolpyruvate
PPP pathway: pentose phosphate pathway
PYR: pyruvate
R5P: ribose 5-phosphate
Ru5P: ribulose 5-phosphate
S7P: sedoheptulose 7-phosphate
SUC: succinate
SucCoA: succinyl-CoA
TCA cycle: tricarboxylic acid cycle
X5P: xylulose 5-phosphate
