# Supplementary material for: 13C-Metabolic Flux Analysis Reveals the Metabolic Flux Redistribution for Enhanced Production of Poly-γ-Glutamic Acid in dlt Over-Expressed Bacillus licheniformis
Source: Front Microbiol. 2019 Feb 1;10:105. doi: 10.3389/fmicb.2019.00105 (PMC6367249; doi:10.3389/fmicb.2019.00105)
Supplement: Supplementary file 1 [file Data_Sheet_1.docx]

**^13^C-Metabolic flux analysis reveals the metabolic flux redistribution for enhanced production of poly-γ-glutamic acid in *dlt* over-expressed *Bacillus licheniformis***

***Penghui He ^1^, Ni Wan ^2^, Dongbo Cai ^1^, Shiying Hu ^1^, Yaozhong Chen ^1^, Shunyi Li ^1^, Shouwen Chen ^1,3*^***

*^1^ State Key Laboratory of Biocatalysis and Enzyme Engineering*, *Environmental Microbial Technology Center of Hubei Province*, *College of Life Sciences, Hubei University, Wuhan 430062,China*

*^2^* *Mechanical Engineering and Materials Science, Washington University, St. Louis, MO, 63130, USA*

*^3^ State Key Laboratory of Agricultural Microbiology, Huazhong Agricultural University, Wuhan 430070, China*

^*^Corresponding author: Prof. Shouwen Chen

Tel./fax.: +86 027-88666081.

*E-mail address*: mel212@126.com (S. Chen).

*Postal address*: 368 Youyi Avenue, Wuchang District, Wuhan 430062, Hubei, PR China

**Table S1 Related primers for plasmids construction**

| Names | 5'→3’ sequence |
| --- | --- |
| △*dltA*-F1 | GC**GAGCTC**ATAGGGGAGGCTCTGAGCT |
| △*dltA*-R1 | ACGATCATGGCAATCAGACTGGAATGTACGGATGGC |
| △*dltA*-F2 | GCCATCCGTACATTCCAGTCTGATTGCCATGATCGT |
| △*dltA*-R2 | GG**TCTAGA**AAAATGTTGCCCAGTTGTC |
| △*dltA*-YF | ATTGTGGGTTCCGCATTT |
| △*dltA*-YR | ATTGCTCCTTCGTCCATA |
| △*dltB*-F1 | GC**GAGCTC**GGCGGTCGTTATTCCATA |
| △*dltB*-R1 | AAGGAACAGCAGCACATAATGTTGCCCAGTTGTCAA |
| △*dltB*-F2 | TTGACAACTGGGCAACATTATGTGCTGCTGTTCCTT |
| △*dltB-*R2 | GG**TCTAGA**AGCGATCTTTCGGAACCAG |
| △*dltB*-YF | ACGCAGGGTACAAAGAAA |
| △*dltB*-YR | AAACCACTGCGGAGAAAT |
| △*dltC*-F1 | GC**GAGCTC**CAGCATCTCACATGGATTG |
| △*dltC*-R1 | CGAATAAAAAACGCTTTTTCATATGAAAATCTCCTTTTAGTGT |
| △*dltC*-F2 | ACACTAAAAGGAGATTTTCATATGAAAAAGCGTTTTTTATTCG |
| △*dltC*-R2 | GG**TCTAGA**AAAGCAGAGCGTCGTTTGT |
| △*dltC*-YF | GGTATGGACGAAGGAGCA |
| △*dltC*-YR | CTTCGGTCCGTTTCCTTC |
| △*dltD*-F1 | GC**GAGCTC**AGAAGCACAAGTGGTGGC |
| △*dltD*-R1 | CAGCCAAGGTGGAGCGTATTACCCTGGAACATCATCG |
| △*dltD*-F2 | CGATGATGTTCCAGGGTAATACGCTCCACCTTGGCTG |
| △*dltD*-R2 | GG**TCTAGA**ACGGTCGTTGGATTTATTG |
| △*dltD*-YF | TAATAAGCCGTTCATCAGC |
| △*dltD*-YR | TTTCACAGGGTGGATGCC |
| P43-F | GC**GAATTC**TGATAGGTGGTATGTTTTCGCT |
| P43-R | GTGTACATTCCTCTCTTACCTA |
| *dltA*-F | ATGAAACTGATAGAAACAATTA |
| *dltA*-R | TCATACGGTTACCTCTTCTTTT |
| TamyL-F | AAGAGCAGAGAGGACGGATTTC |
| TamyL-R | GG**TCTAGA**CGCAATAATGCCGTCGCACTGG |
| *dltB*-F | ATGACACCCTATGGTTCATTTC |
| *dltB*-R | TTAGTGTATTAGTTTCCCTGAG |
| *dltC*-F | ATGGATTTTAATCAAGAAGTAC |
| *dltC*-R | TTATCTCAACTCATTCAGCTGC |
| *dltD*-F | ATGAAAAAGCGTTTTTTATTCG |
| *dltD*-R | TTATGATGACTTTTTATAAAAG |
| T2-F | ATGTGATAACTCGGCGTA |
| T2-R | GCAAGCAGCAGATTACGC |
| pHY-F | GTTTATTATCCATACCCTTAC |
| pHY-R | CAGATTTCGTGATGCTTGTC |

Note: The red color nucleotide bases indicated the restriction endonuclease sites.

**Table S2 The primers used for RT-qPCR in this study**

| Names | 5'→3’ sequence |
| --- | --- |
| 16s-RT-F | ACCTAACCAGAAAGCCACGG |
| 16s-RT-R | GTTTACGGCGTGGACTACCA |
| *ptsG*-RT-F | AACGGGAGTATTCGGGGGTA |
| *ptsG*-RT-R | CCCAGCAGCAATGCACAAAT |
| *glcU*-RT-F | CTCGTGTATGTCGTCGTCGT |
| *glcU*-RT-R | TCAGCTGCCGCTTTGTTTTC |
| *glcP*-RT-F | TTCTAAACGGCGTCCTGCTT |
| *glcP*-RT-R | GCCAGGCATATCGTTCCGTA |
| *zwf*-RT-F | GCCGATCAAGCTCGACTACT |
| *zwf*-RT-R | CCATAGAGCCCGCCTCATAGT |
| *gndA*-RT-F | AGCTTGAAGATTTCGTACAG |
| *gndA*-RT-R | TCGCCGCCGGAAATGCCGAT |
| *pgi*-RT-F | TCGGCAACAACATCAGCTCT |
| *pgi*-RT-R | GCAAGCGTTTTTAATGCGCC |
| *gapA*-RT-F | CAGCTCCTGCGAGTGAAGAA |
| *gapA*-RT-R | GACGGTAGTCTTTGTGCGGA |
| *citA*-RT-F | GAGCTGCTCGCTTCATTTCG |
| *citA*-RT-R | CTTCTGTCGGACGCTTTCCT |
| *citB*-RT-F | GAATTCTTCGGTCCGGGTGT |
| *citB*-RT-R | GAGATCCTGAGGACGCTTCG |
| *icd*-RT-F | CATCAAAGACAGCATCGCCG |
| *icd*-RT-R | TGTGACGACTTTGGATGCGA |
| *rocG*-RT-F | AGTATGTCAGCGAGCATCCG |
| *rocG*-RT-R | CGGTCCGAATAGAACTCCCG |
| *gltA*-RT-F | TTGCAATGAACCGTCTCGGA |
| *gltA*-RT-R | GATCCGCGTACATTGGCAAC |
| *pgsB*-RT-F | ATCAAAAGAAAGCCGCAAGG |
| *pgsB*-RT-R | AGTCGGTCCCATCACATCC |
| *pgsC*-RT-F | GCTGCTCGTTCTTTTTGTC |
| *pgsC*-RT-R | CGTACTTCCAAGCGTAATCG |
| *pgdS*-RT-F | TCAGGCATATCGGCGAAGTC |
| *pgdS*-RT-R | CAATAGGCGCTCGTCTCCAT |
| *comA*-RT-F | TGATCATCCGGCTGTCATGG |
| *comA*-RT-R | ATTCCGTTTTGCTGATGGCG |
| *comP*-RT-F | CCGACATCGGCAAAATCGTC |
| *comP*-RT-R | GGAGATCGCGGGCTAAATCA |
| *degU*-RT-F | GATCAGCGTATCGGCATCC |
| *degU*-RT-R | TTTGAGGTAGTGGCCGAAGG |
| *degS*-RT-F | GAGACAGGCTGGAAGTGCAT |
| *degS*-RT-R | TTTCCCGCTGCTGGATCATT |

**Table S3 Experimentally measured and simulated mass isotopomer distributions (mol%) of amino acids fragment from *B. licheniformis* WX-02/pHY300 and WX-02/pHY-*dltB* in the mid-logarithmic.** Cultures were grown with [1,2-^13^C] glucose. Three biological replicates were used for isotopomer analysis.

| **Strains** | **WX-02/pHY300** | | | **WX-02/pHY-dltB** | | |
| --- | --- | --- | --- | --- | --- | --- |
| **Ion Fragments** | **Measured data** | **Standard deviation** | **Simulated data** | **Measured data** | **Standard deviation** | **Simulated data** |
| **Alanine [M-57]'** |  |  |  |  |  |  |
| M0 | 0.5260 | 0.0086 | 0.5265 | 0.5241 | 0.004 | 0.5181 |
| M1 | 0.0724 | 0.0012 | 0.0763 | 0.0705 | 0.0108 | 0.0857 |
| M2 | 0.3935 | 0.0089 | 0.3710 | 0.4028 | 0.0075 | 0.3498 |
| M3 | 0.0081 | 0.0008 | 0.0262 | 0.0026 | 0.0031 | 0.0463 |
| **Alanine [M-85]'** |  |  |  |  |  |  |
| M0 | 0.5333 | 0.0069 | 0.5430 | 0.5341 | 0.0011 | 0.5356 |
| M1 | 0.1254 | 0.0031 | 0.1283 | 0.1162 | 0.0130 | 0.1346 |
| M2 | 0.3413 | 0.0049 | 0.3287 | 0.3497 | 0.0141 | 0.3298 |
| **Alanine [M-159]'** |  |  |  |  |  |  |
| M0 | 0.533 | 0.0059 | 0.5430 | 0.5361 | 0.0014 | 0.5356 |
| M1 | 0.1177 | 0.0013 | 0.1283 | 0.1143 | 0.0144 | 0.1346 |
| M2 | 0.3492 | 0.0073 | 0.3287 | 0.3496 | 0.0140 | 0.3298 |
| **Glycine [M-57]'** |  |  |  |  |  |  |
| M0 | 0.5948 | 0.0005 | 0.5747 | 0.6047 | 0.0375 | 0.5516 |
| M1 | 0.4018 | 0.0035 | 0.3856 | 0.3962 | 0.0053 | 0.3745 |
| M2 | 0.0034 | 0.0039 | 0.0397 | 0 | 0.0052 | 0.0739 |
| **Glycine [M-85]'** |  |  |  |  |  |  |
| M0 | 0.6435 | 0.0192 | 0.6299 | 0.6658 | 0.0112 | 0.6357 |
| M1 | 0.3565 | 0.0192 | 0.3701 | 0.3342 | 0.0013 | 0.3643 |
| **Glycine [M-159]'** |  |  |  |  |  |  |
| M0 | 0.6143 | 0.0107 | 0.6299 | 0.6548 | 0.0105 | 0.6357 |
| M1 | 0.3857 | 0.0107 | 0.3701 | 0.3452 | 0.0035 | 0.3643 |
| **Valine [M-57]'** |  |  |  |  |  |  |
| M0 | 0.3114 | 0.0067 | 0.2859 | 0.3097 | 0.0072 | 0.2775 |
| M1 | 0.0952 | 0.0046 | 0.1090 | 0.107 | 0.0062 | 0.1157 |
| M2 | 0.39 | 0.0095 | 0.3843 | 0.3748 | 0.0035 | 0.3698 |
| M3 | 0.0698 | 0.0052 | 0.0869 | 0.0694 | 0.0178 | 0.1002 |
| M4 | 0.129 | 0.0111 | 0.1253 | 0.1347 | 0.0093 | 0.1216 |
| M5 | 0.0046 | 0.0047 | 0.0086 | 0.0044 | 0.0077 | 0.0153 |
| **Valine [M-159]'** |  |  |  |  |  |  |
| M0 | 0.3067 | 0.0043 | 0.2949 | 0.3124 | 0.0101 | 0.2868 |
| M1 | 0.1184 | 0.0010 | 0.1393 | 0.1225 | 0.0103 | 0.1442 |
| M2 | 0.3565 | 0.0062 | 0.3734 | 0.3573 | 0.0010 | 0.3714 |
| M3 | 0.0923 | 0.0035 | 0.0843 | 0.0931 | 0.0030 | 0.0888 |
| M4 | 0.1261 | 0.0059 | 0.1080 | 0.1147 | 0.0042 | 0.1088 |
| **Leucine [M-15]'** |  |  |  |  |  |  |
| M0 | 0.1997 | 0.0115 | 0.1601 | 0.2165 | 0.0045 | 0.1523 |
| M1 | 0.1082 | 0.0011 | 0.1135 | 0.0975 | 0.0172 | 0.1218 |
| M2 | 0.3167 | 0.0230 | 0.3176 | 0.2939 | 0.0108 | 0.3092 |
| M3 | 0.1312 | 0.0075 | 0.1395 | 0.1401 | 0.0074 | 0.1506 |
| M4 | 0.1809 | 0.0031 | 0.1922 | 0.1739 | 0.0096 | 0.1874 |
| M5 | 0.0282 | 0.0060 | 0.0416 | 0.0445 | 0.0002 | 0.0448 |
| M6 | 0.035 | 0.0039 | 0.0355 | 0.0336 | 0.0002 | 0.0339 |
| **Leucine [M-159]'** |  |  |  |  |  |  |
| M0 | 0.1973 | 0.0112 | 0.1768 | 0.2226 | 0.0118 | 0.1776 |
| M1 | 0.1859 | 0.0028 | 0.2016 | 0.1838 | 0.0104 | 0.1985 |
| M2 | 0.2609 | 0.0105 | 0.2797 | 0.2478 | 0.0262 | 0.2849 |
| M3 | 0.2019 | 0.0023 | 0.2001 | 0.1973 | 0.0006 | 0.1964 |
| M4 | 0.1026 | 0.0035 | 0.0985 | 0.0994 | 0.0013 | 0.1012 |
| M5 | 0.0513 | 0.0023 | 0.0432 | 0.0492 | 0.0056 | 0.0414 |
| **Serine [M-57]'** |  |  |  |  |  |  |
| M0 | 0.4984 | 0.0004 | 0.5266 | 0.4937 | 0.0231 | 0.5264 |
| M1 | 0.1099 | 0.0249 | 0.1266 | 0.1357 | 0.0027 | 0.1396 |
| M2 | 0.3868 | 0.0175 | 0.3234 | 0.3717 | 0.0545 | 0.2946 |
| M3 | 0.0049 | 0.0069 | 0.0233 | 0 | 0.0279 | 0.0394 |
| **Serine [M-159]'** |  |  |  |  |  |  |
| M0 | 0.5215 | 0.0143 | 0.5415 | 0.513 | 0.0202 | 0.5416 |
| M1 | 0.163 | 0.0081 | 0.1410 | 0.1549 | 0.0076 | 0.1441 |
| M2 | 0.3155 | 0.0062 | 0.3175 | 0.3321 | 0.0127 | 0.3142 |
| **Theronine [M-57]'** |  |  |  |  |  |  |
| M0 | 0.3869 | 0.0101 | 0.4226 | 0.3911 | 0.0128 | 0.4315 |
| M1 | 0.1769 | 0.0272 | 0.1688 | 0.1873 | 0.0111 | 0.1716 |
| M2 | 0.3166 | 0.0284 | 0.3005 | 0.3457 | 0.0445 | 0.2821 |
| M3 | 0.1092 | 0.0200 | 0.0901 | 0.0523 | 0.0301 | 0.0949 |
| M4 | 0.0103 | 0.0086 | 0.0180 | 0.0236 | 0.0027 | 0.0198 |
| **Theronine [M-85]'** |  |  |  |  |  |  |
| M0 | 0.4128 | 0.0068 | 0.4389 | 0.4278 | 0.0124 | 0.4454 |
| M1 | 0.1879 | 0.01860 | 0.1969 | 0.2334 | 0.0196 | 0.1882 |
| M2 | 0.3123 | 0.02821 | 0.2878 | 0.2489 | 0.0308 | 0.2925 |
| M3 | 0.0869 | 0.0029 | 0.0764 | 0.0898 | 0.0113 | 0.0738 |
| **Phenylalanine f302'** |  |  |  |  |  |  |
| M0 | 0.6779 | 0.0011 | 0.6039 | 0.7001 | 0.0145 | 0.6231 |
| M1 | 0.3178 | 0.0035 | 0.3689 | 0.3039 | 0.0214 | 0.3342 |
| M2 | 0.0043 | 0.0024 | 0.0272 | 0 | 0.0301 | 0.0426 |
| **Asparate [M-57]'** |  |  |  |  |  |  |
| M0 | 0.3993 | 0.0005 | 0.4226 | 0.4182 | 0.0094 | 0.4315 |
| M1 | 0.1702 | 0.0044 | 0.1688 | 0.1728 | 0.0009 | 0.1716 |
| M2 | 0.3248 | 0.0143 | 0.3005 | 0.2927 | 0.0075 | 0.2821 |
| M3 | 0.0939 | 0.0114 | 0.0901 | 0.1065 | 0.0082 | 0.0949 |
| M4 | 0.0118 | 0.0010 | 0.0180 | 0.0097 | 0.0071 | 0.0198 |
| **Asparate [M-159]'** |  |  |  |  |  |  |
| M0 | 0.4188 | 0.0112 | 0.4389 | 0.4286 | 0.0119 | 0.4454 |
| M1 | 0.2153 | 0.0010 | 0.1969 | 0.2033 | 0.0107 | 0.1882 |
| M2 | 0.2894 | 0.0010 | 0.2878 | 0.2845 | 0.0057 | 0.2925 |
| M3 | 0.0765 | 0.0112 | 0.0764 | 0.0837 | 0.0070 | 0.0738 |
| **Asparate f302'** |  |  |  |  |  |  |
| M0 | 0.5818 | 0.0032 | 0.6039 | 0.604 | 0.0071 | 0.6141 |
| M1 | 0.3564 | 0.0086 | 0.3689 | 0.3437 | 0.0094 | 0.357 |
| M2 | 0.0618 | 0.0054 | 0.0272 | 0.0523 | 0.0165 | 0.029 |
| **Glutamate [M-57]'** |  |  |  |  |  |  |
| M0 | 0.2347 | 0.0029 | 0.2384 | 0.2361 | 0.0003 | 0.2365 |
| M1 | 0.1535 | 0.0047 | 0.1632 | 0.1545 | 0.0111 | 0.1702 |
| M2 | 0.3255 | 0.0013 | 0.3258 | 0.3213 | 0.0017 | 0.3237 |
| M3 | 0.1564 | 0.0052 | 0.1431 | 0.1493 | 0.0037 | 0.144 |
| M4 | 0.1069 | 0.0062 | 0.1044 | 0.1145 | 0.0083 | 0.1027 |
| M5 | 0.023 | 0.0006 | 0.0251 | 0.0243 | 0.0010 | 0.023 |
| **Glutamate [M-159]'** |  |  |  |  |  |  |
| M0 | 0.2974 | 0.0004 | 0.2952 | 0.3029 | 0.0089 | 0.2903 |
| M1 | 0.1362 | 0.0079 | 0.1447 | 0.1406 | 0.0103 | 0.1551 |
| M2 | 0.3685 | 0.0011 | 0.3693 | 0.3609 | 0.0011 | 0.3625 |
| M3 | 0.0866 | 0.0103 | 0.0862 | 0.0899 | 0.0011 | 0.0914 |
| M4 | 0.1113 | 0.0032 | 0.1047 | 0.1058 | 0.0035 | 0.1008 |
| **Histidine [M-57]'** |  |  |  |  |  |  |
| M0 | 0.2278 | 0.0189 | 0.2111 | 0.2296 | 0.0309 | 0.1859 |
| M1 | 0.3525 | 0.0061 | 0.4445 | 0.347 | 0.0287 | 0.3876 |
| M2 | 0.2931 | 0.0148 | 0.2499 | 0.3095 | 0.0404 | 0.2524 |
| M3 | 0.0665 | 0.0103 | 0.0588 | 0.0589 | 0.0163 | 0.0820 |
| M4 | 0.0606 | 0.0140 | 0.0284 | 0.0563 | 0.0023 | 0.0595 |
| M5 | 0.0022 | 0.0015 | 0.0070 | 0.0000 | 0.0209 | 0.0296 |
| M6 | 0.0000 | 0.0032 | 0.0004 | 0.0004 | 0.0018 | 0.0030 |

**Table S4 The complete list of flux values and exchange coffecients in the central metabolism estimated from ^13^C MFA model.** The estimated intracellular and exchange fluxes were normalized to glucose uptake rate of 100. Accurate 95% confidence intervals of fluxes were determined by evaluating the sensitivity of the minimized SSR to flux variations.

1. Flux values of the control strain WX-02/pHY300

| **Reactions** | **Best fit** | **95% confidence interval lower bounds** | **95% confidence interval upper bounds** | **Standard deviations** |
| --- | --- | --- | --- | --- |
| **INTRACELLULAR FLUXES** | | | | |
| Gluc.ext + ATP -> G6P | 100.00 | 97.83 | 102.19 | 1.29 |
| G6P <-> F6P | 65.37 | 63.27 | 67.45 | 1.93 |
| F6P + ATP -> FBP | 84.57 | 82.45 | 86.72 | 1.26 |
| FBP <-> DHAP + GAP | 84.57 | 82.45 | 86.72 | 1.26 |
| DHAP <-> GAP | 84.57 | 82.45 | 86.72 | 1.26 |
| GAP <-> 3PG + ATP + NADH | 177.83 | 173.54 | 182.16 | 2.48 |
| 3PG <-> PEP | 170.54 | 166.24 | 174.87 | 2.51 |
| PEP -> Pyr + ATP | 167.30 | 163.01 | 172.47 | 3.27 |
| G6P -> 6PG + NADPH | 33.77 | 33.25 | 34.35 | 1.83 |
| 6PG -> Ru5P + CO2 + NADPH | 33.77 | 33.25 | 34.35 | 1.83 |
| Ru5P <-> X5P | 19.50 | 19.16 | 19.89 | 1.22 |
| Ru5P <-> R5P | 14.27 | 14.10 | 14.46 | 0.61 |
| X5P + R5P <-> GAP + S7P | 10.51 | 10.33 | 10.70 | 0.61 |
| GAP + S7P <-> E4P + F6P | 10.51 | 10.33 | 10.70 | 0.61 |
| X5P + E4P <-> GAP + F6P | 9.00 | 8.82 | 9.19 | 0.61 |
| Pyr -> AcCoA + CO2 + NADH | 74.99 | 71.38 | 78.96 | 2.47 |
| OAC + AcCoA -> Cit | 13.86 | 11.25 | 16.49 | 2.44 |
| Cit <-> ICit | 13.86 | 11.25 | 16.49 | 2.44 |
| ICit <-> AKG + CO2 + NADPH | 13.58 | 11.05 | 16.12 | 2.22 |
| AKG -> SucCoA + CO2 + NADH | 1.29 | 0.19 | 2.32 | 2.07 |
| SucCoA <-> Suc + ATP | 0.68 | 0.34 | 1.79 | 2.07 |
| Suc <-> Fum + FADH2 | 1.57 | 0.79 | 2.52 | 2.33 |
| Fum <-> Mal | 3.12 | 2.34 | 4.07 | 2.33 |
| Mal <-> OAC + NADH | 3.40 | 2.24 | 4.80 | 0.44 |
| ICit -> Glyox + Suc | 0.28 | 0.00 | 1.25 | 0.05 |
| Glyox + AcCoA -> Mal | 0.28 | 0.00 | 1.25 | 0.05 |
| Mal -> Pyr + CO2 + NADPH | 0.00 | 0.00 | 0.42 | 1.23 |
| Mal -> Pyr + CO2 + NADH | 0.00 | 0.00 | 1.19 | 1.23 |
| Pyr + CO2 -> OAC | 19.49 | 17.12 | 23.98 | 2.93 |
| OAC + ATP -> PEP + CO2 | 0.00 | 0.00 | 4.08 | 3.25 |
| AKG + NADPH -> Glu | 33.07 | 30.66 | 35.50 | 1.23 |
| Glu + ATP -> Gln | 2.83 | 2.83 | 2.83 | 0.00 |
| Glu + ATP + 2*NADPH -> Pro | 0.88 | 0.88 | 0.88 | 0.00 |
| Glu + CO2 + Gln + Asp + AcCoA + 5*ATP + NADPH -> Arg + AKG + Fum + Ac | 1.18 | 1.18 | 1.18 | 0.00 |
| OAC + Glu -> Asp + AKG | 7.61 | #VALUE! | 8.00 | 0.90 |
| Asp + 2*ATP -> Asn | 0.96 | 0.96 | 0.96 | 0.00 |
| Pyr + NADH + NH3 -> Ala | 2.04 | 2.04 | 2.66 | 1.23 |
| Ala + AKG -> Pyr + Glu | 0.00 | 0.00 | 0.62 | 1.23 |
| 3PG + Glu + NADPH -> Ser + AKG + NADH | 4.70 | 4.51 | 4.79 | 0.45 |
| Ser <-> Gly + MEETHF | 2.64 | 2.45 | 2.72 | 0.45 |
| Gly <-> CO2 + MEETHF + NADH | 0.20 | 0.18 | 0.39 | 0.45 |
| Thr -> Gly + AcCoA + NADH | 0.00 | 0.00 | 0.38 | 0.90 |
| Ser + AcCoA + 3*ATP + 4*NADPH -> Cys + Ac | 0.98 | 0.98 | 0.98 | 0.00 |
| Asp + Pyr + Glu + SucCoA + ATP + 2*NADPH -> LLDAP + AKG + Suc | 1.37 | 1.37 | 1.37 | 0.00 |
| LLDAP -> Lys + CO2 | 1.37 | 1.37 | 1.37 | 0.00 |
| Asp + 2*ATP + 2*NADPH -> Thr | 2.16 | 1.82 | 2.55 | 0.90 |
| Asp + METHF + Cys + SucCoA + ATP + 2*NADPH -> Met + Pyr + Suc | 0.61 | 0.61 | 0.61 | 0.00 |
| Pyr + Pyr + Glu + NADPH -> Val + CO2 + AKG | 1.68 | 1.68 | 1.68 | 0.00 |
| AcCoA + Pyr + Pyr + Glu + NADPH -> Leu + CO2 + CO2 + AKG + NADH | 1.79 | 1.79 | 1.79 | 0.00 |
| Thr + Pyr + Glu + NADPH -> Ile + CO2 + AKG | 1.16 | 1.16 | 1.16 | 0.00 |
| PEP + PEP + E4P + Glu + ATP + NADPH -> Phe + CO2 + AKG | 0.74 | 0.74 | 0.74 | 0.00 |
| PEP + PEP + E4P + Glu + ATP + NADPH -> Tyr + CO2 + AKG + NADH | 0.55 | 0.55 | 0.55 | 0.00 |
| Ser + R5P + PEP + E4P + PEP + Gln + 3*ATP + NADPH -> Trp + CO2 + GAP + Pyr + Glu | 0.23 | 0.23 | 0.23 | 0.00 |
| R5P + FTHF + Gln + Asp + 5*ATP -> His + AKG + Fum + 2*NADH | 0.38 | 0.38 | 0.38 | 0.00 |
| MEETHF + NADH -> METHF | 0.61 | 0.61 | 0.61 | 0.00 |
| MEETHF -> FTHF + NADPH | 0.38 | 0.38 | 0.38 | 0.00 |
| NADH -> 3*ATP | 253.93 | 249.92 | 261.74 | 7.78 |
| FADH2 -> 2*ATP | 1.57 | 0.79 | 2.52 | 2.33 |
| ATP -> ATP.ext | 784.05 | 769.89 | 810.89 | 22.52 |
| Ac -> Ac.ext | 48.72 | 46.03 | 51.42 | 1.38 |
| CO2 -> CO2.ext | 174.26 | 169.71 | 178.83 | 6.35 |
| 0.488*Ala + 0.281*Arg + 0.229*Asn + 0.229*Asp + 0.087*Cys + 0.25*Glu + 0.25*Gln + 0.582*Gly + 0.09*His + 0.276*Ile + 0.428*Leu + 0.326*Lys + 0.146*Met + 0.176*Phe + 0.21*Pro + 0.205*Ser + 0.241*Thr + 0.054*Trp + 0.131*Tyr + 0.402*Val + 0.205*G6P + 0.071*F6P + 0.754*R5P + 0.129*GAP + 0.619*3PG + 0.051*PEP + 0.083*Pyr + 2.51*AcCoA + 0.087*AKG + 0.34*OAC + 0.443*MEETHF + 33.25*ATP + 5.363*NADPH -> 39.68*Biomass + 1.455*NADH | 4.19 | 4.19 | 4.19 | 0.00 |
| CO2.unlabeled + CO2 -> CO2 + CO2.out | 51.10 | 30.19 | 83.96 | 25.99 |
| AcCoA <-> Ac + ATP | 46.57 | 43.88 | 49.26 | 1.38 |
| Pyr + Pyr -> Acetoin + CO2 + CO2 | 30.89 | 28.01 | 33.19 | 1.23 |
| Acetoin + NADH <-> BDO | 15.11 | 12.70 | 17.52 | 1.27 |
| Acetoin -> AcCoA + AcCoA + NADH | 0.09 | 0.00 | 2.69 | 1.78 |
| Acetoin + ATP -> Acetoin_ex | 15.70 | 13.29 | 18.11 | 1.27 |
| BDO -> BDO_ex | 15.11 | 12.70 | 17.52 | 1.27 |
| Glu + ATP -> PGA | 7.77 | 5.36 | 10.19 | 1.26 |
| PGA -> PGA_ex | 7.77 | 5.36 | 10.19 | 1.26 |
| **EXCHANGE FLUXES** | |  |  |  |
| G6P <-> F6P | 0.00 | 0.00 | 5.27 | 10.32 |
| FBP <-> DHAP + GAP | >1000 | 0.00 | >1000 | >1000 |
| DHAP <-> GAP | 88.62 | 0.00 | >1000 | >1000 |
| GAP <-> 3PG + ATP + NADH | 0.00 | 0.00 | >1000 | >1000 |
| 3PG <-> PEP | 0.00 | 0.00 | >1000 | >1000 |
| Ru5P <-> X5P | 10.98 | 7.91 | 14.12 | 4.15 |
| Ru5P <-> R5P | 57.44 | 36.63 | 98.74 | 22.72 |
| X5P + R5P <-> GAP + S7P | 26.28 | 23.19 | 513.73 | 2.39 |
| GAP + S7P <-> E4P + F6P | >1000 | 28.00 | >1000 | >1000 |
| X5P + E4P <-> GAP + F6P | 0.00 | 0.00 | 0.20 | 1.51 |
| Cit <-> ICit | 0.00 | 0.00 | >1000 | >1000 |
| ICit <-> AKG + CO2 + NADPH | 0.00 | 0.00 | >1000 | 334.81 |
| SucCoA <-> Suc + ATP | 5.11 | 0.00 | >1000 | >1000 |
| Suc <-> Fum + FADH2 | 21.64 | 0.00 | >1000 | >1000 |
| Fum <-> Mal | >1000 | 0.00 | >1000 | >1000 |
| Mal <-> OAC + NADH | 0.00 | 0.00 | >1000 | 4.94 |
| Ser <-> Gly + MEETHF | 0.54 | 0.36 | 0.74 | 0.11 |
| Gly <-> CO2 + MEETHF + NADH | 0.00 | 0.00 | 0.23 | 0.19 |
| AcCoA <-> Ac + ATP | 64.84 | 0.00 | >1000 | >1000 |
| Acetoin + NADH <-> BDO | 11.06 | 0.00 | >1000 | >1000 |

1. Flux values of the recombinant strain WX-02/pHY-*dltB*

| **Reactions** | **Best fit** | **95% confidence interval lower bounds** | **95% confidence interval upper bounds** | **Standard deviations** |
| --- | --- | --- | --- | --- |
| **INTRACELLULAR FLUXES** | | | | |
| Gluc.ext + ATP -> G6P | 100.00 | 97.81 | 102.25 | 1.29 |
| G6P <-> F6P | 62.67 | 60.65 | 64.73 | 2.08 |
| F6P + ATP -> FBP | 83.38 | 81.26 | 85.54 | 1.27 |
| FBP <-> DHAP + GAP | 83.38 | 81.26 | 85.54 | 1.27 |
| DHAP <-> GAP | 83.38 | 81.26 | 85.54 | 1.27 |
| GAP <-> 3PG + ATP + NADH | 176.12 | 171.82 | 180.52 | 2.47 |
| 3PG <-> PEP | 168.27 | 163.97 | 169.53 | 2.51 |
| PEP -> Pyr + ATP | 164.78 | 160.48 | 169.20 | 3.03 |
| G6P -> 6PG + NADPH | 36.41 | 35.74 | 37.13 | 2.05 |
| 6PG -> Ru5P + CO2 + NADPH | 36.41 | 35.74 | 37.13 | 2.05 |
| Ru5P <-> X5P | 21.03 | 20.59 | 21.51 | 1.37 |
| Ru5P <-> R5P | 15.38 | 15.16 | 15.62 | 0.68 |
| X5P + R5P <-> GAP + S7P | 11.33 | 11.11 | 11.57 | 0.68 |
| GAP + S7P <-> E4P + F6P | 11.33 | 11.11 | 11.57 | 0.68 |
| X5P + E4P <-> GAP + F6P | 9.70 | 9.48 | 9.94 | 0.68 |
| Pyr -> AcCoA + CO2 + NADH | 83.33 | 0.00 | 87.86 | 3.49 |
| OAC + AcCoA -> Cit | 19.18 | 16.45 | 22.00 | 2.33 |
| Cit <-> ICit | 19.18 | 16.45 | 22.00 | 2.33 |
| ICit <-> AKG + CO2 + NADPH | 18.44 | 15.88 | 21.03 | 1.92 |
| AKG -> SucCoA + CO2 + NADH | 1.31 | 0.00 | 2.62 | 1.66 |
| SucCoA <-> Suc + ATP | 0.82 | 0.50 | 2.13 | 1.66 |
| Suc <-> Fum + FADH2 | 2.05 | 0.92 | 3.23 | 2.20 |
| Fum <-> Mal | 3.72 | 2.59 | 4.90 | 2.20 |
| Mal <-> OAC + NADH | 4.47 | -3.86 | 7.12 | 2.49 |
| ICit -> Glyox + Suc | 0.75 | 0.00 | 2.54 | 1.14 |
| Glyox + AcCoA -> Mal | 0.75 | 0.00 | 2.54 | 1.14 |
| Mal -> Pyr + CO2 + NADPH | 0.00 | 0.00 | 0.49 | 1.74 |
| Mal -> Pyr + CO2 + NADH | 0.00 | 0.00 | 8.38 | 1.74 |
| Pyr + CO2 -> OAC | 24.44 | 21.86 | 32.92 | 1.96 |
| OAC + ATP -> PEP + CO2 | 0.00 | 0.00 | 3.19 | 3.02 |
| AKG + NADPH -> Glu | 39.51 | 37.10 | 41.86 | 1.74 |
| Glu + ATP -> Gln | 3.04 | 3.04 | 3.04 | 0.00 |
| Glu + ATP + 2*NADPH -> Pro | 0.95 | 0.95 | 0.95 | 0.00 |
| Glu + CO2 + Gln + Asp + AcCoA + 5*ATP + NADPH -> Arg + AKG + Fum + Ac | 1.27 | 1.27 | 1.27 | 0.00 |
| OAC + Glu -> Asp + AKG | 8.20 | 7.88 | 8.68 | 1.11 |
| Asp + 2*ATP -> Asn | 1.03 | 1.03 | 1.03 | 0.00 |
| Pyr + NADH + NH3 -> Ala | 2.20 | 2.20 | 2.73 | 1.74 |
| Ala + AKG -> Pyr + Glu | 0.00 | 0.00 | 0.53 | 1.74 |
| 3PG + Glu + NADPH -> Ser + AKG + NADH | 5.06 | 4.82 | 5.52 | 0.55 |
| Ser <-> Gly + MEETHF | 2.84 | 2.60 | 3.08 | 0.55 |
| Gly <-> CO2 + MEETHF + NADH | 0.22 | 0.00 | 0.46 | 0.55 |
| Thr -> Gly + AcCoA + NADH | 0.00 | 0.00 | 0.49 | 1.11 |
| Ser + AcCoA + 3*ATP + 4*NADPH -> Cys + Ac | 1.05 | 1.05 | 1.05 | 0.00 |
| Asp + Pyr + Glu + SucCoA + ATP + 2*NADPH -> LLDAP + AKG + Suc | 1.47 | 1.47 | 1.47 | 0.00 |
| LLDAP -> Lys + CO2 | 1.47 | 1.47 | 1.47 | 0.00 |
| Asp + 2*ATP + 2*NADPH -> Thr | 2.33 | 1.93 | 2.82 | 1.11 |
| Asp + METHF + Cys + SucCoA + ATP + 2*NADPH -> Met + Pyr + Suc | 0.66 | 0.66 | 0.66 | 0.00 |
| Pyr + Pyr + Glu + NADPH -> Val + CO2 + AKG | 1.81 | 1.81 | 1.81 | 0.00 |
| AcCoA + Pyr + Pyr + Glu + NADPH -> Leu + CO2 + CO2 + AKG + NADH | 1.93 | 1.93 | 1.93 | 0.00 |
| Thr + Pyr + Glu + NADPH -> Ile + CO2 + AKG | 1.24 | 1.24 | 1.24 | 0.00 |
| PEP + PEP + E4P + Glu + ATP + NADPH -> Phe + CO2 + AKG | 0.79 | 0.79 | 0.79 | 0.00 |
| PEP + PEP + E4P + Glu + ATP + NADPH -> Tyr + CO2 + AKG + NADH | 0.59 | 0.59 | 0.59 | 0.00 |
| Ser + R5P + PEP + E4P + PEP + Gln + 3*ATP + NADPH -> Trp + CO2 + GAP + Pyr + Glu | 0.24 | 0.24 | 0.24 | 0.00 |
| R5P + FTHF + Gln + Asp + 5*ATP -> His + AKG + Fum + 2*NADH | 0.41 | 0.41 | 0.41 | 0.00 |
| MEETHF + NADH -> METHF | 0.66 | 0.66 | 0.66 | 0.00 |
| MEETHF -> FTHF + NADPH | 0.41 | 0.41 | 0.41 | 0.00 |
| NADH -> 3*ATP | 262.64 | 258.51 | 272.02 | 11.74 |
| FADH2 -> 2*ATP | 2.05 | 0.92 | 3.23 | 2.20 |
| ATP -> ATP.ext | 800.63 | 749.55 | 832.60 | 28.35 |
| Ac -> Ac.ext | 50.36 | 47.67 | 53.05 | 1.38 |
| CO2 -> CO2.ext | 169.14 | 163.98 | 174.43 | 6.33 |
| 0.488*Ala + 0.281*Arg + 0.229*Asn + 0.229*Asp + 0.087*Cys + 0.25*Glu + 0.25*Gln + 0.582*Gly + 0.09*His + 0.276*Ile + 0.428*Leu + 0.326*Lys + 0.146*Met + 0.176*Phe + 0.21*Pro + 0.205*Ser + 0.241*Thr + 0.054*Trp + 0.131*Tyr + 0.402*Val + 0.205*G6P + 0.071*F6P + 0.754*R5P + 0.129*GAP + 0.619*3PG + 0.051*PEP + 0.083*Pyr + 2.51*AcCoA + 0.087*AKG + 0.34*OAC + 0.443*MEETHF + 33.25*ATP + 5.363*NADPH -> 39.68*Biomass + 1.455*NADH | 4.51 | 4.51 | 4.51 | 0.00 |
| CO2.unlabeled + CO2 -> CO2 + CO2.out | 80.32 | 50.10 | 134.25 | 31.78 |
| AcCoA <-> Ac + ATP | 48.04 | 45.35 | 50.74 | 1.38 |
| Pyr + Pyr -> Acetoin + CO2 + CO2 | 22.57 | 19.78 | 25.24 | 2.72 |
| Acetoin + NADH <-> BDO | 15.01 | 12.60 | 17.42 | 1.27 |
| Acetoin -> AcCoA + AcCoA + NADH | 0.10 | 0.00 | 2.38 | 2.18 |
| Acetoin + ATP -> Acetoin_ex | 7.46 | 5.05 | 9.87 | 1.27 |
| BDO -> BDO_ex | 15.01 | 12.60 | 17.42 | 1.27 |
| Glu + ATP -> PGA | 12.27 | 9.85 | 14.69 | 1.26 |
| PGA -> PGA_ex | 12.27 | 9.85 | 14.69 | 1.26 |
| **EXCHANGE FLUXES** | | | | |
| G6P <-> F6P | 0.00 | 0.00 | 4.01 | 10.37 |
| FBP <-> DHAP + GAP | 526.20 | 0.00 | >1000 | >1000 |
| DHAP <-> GAP | 24.20 | 0.00 | >1000 | >1000 |
| GAP <-> 3PG + ATP + NADH | 0.00 | 0.00 | >1000 | >1000 |
| 3PG <-> PEP | 0.00 | 0.00 | >1000 | >1000 |
| Ru5P <-> X5P | 8.46 | 5.90 | 11.23 | 4.15 |
| Ru5P <-> R5P | 54.19 | 36.04 | 86.76 | 19.76 |
| X5P + R5P <-> GAP + S7P | 31.90 | 28.25 | 74.08 | 2.95 |
| GAP + S7P <-> E4P + F6P | >1000 | 65.13 | >1000 | >1000 |
| X5P + E4P <-> GAP + F6P | 0.00 | 0.00 | 0.18 | 1.64 |
| Cit <-> ICit | 0.16 | 0.00 | >1000 | >1000 |
| ICit <-> AKG + CO2 + NADPH | 0.00 | 0.00 | >1000 | 235.09 |
| SucCoA <-> Suc + ATP | 4.59 | 0.00 | >1000 | >1000 |
| Suc <-> Fum + FADH2 | 0.32 | 0.00 | >1000 | >1000 |
| Fum <-> Mal | 0.00 | 0.00 | >1000 | 6.05 |
| Mal <-> OAC + NADH | >1000 | 0.00 | >1000 | >1000 |
| Ser <-> Gly + MEETHF | 0.64 | 0.43 | 0.86 | 0.14 |
| Gly <-> CO2 + MEETHF + NADH | 0.00 | 0.00 | 0.13 | 0.21 |
| AcCoA <-> Ac + ATP | 12.97 | 0.00 | >1000 | >1000 |
| Acetoin + NADH <-> BDO | 9.43 | 0.00 | >1000 | >1000 |

**Fig. S1 Growth curves of *B. licheniformis* WX-02 and WX-02△*dltB*.**


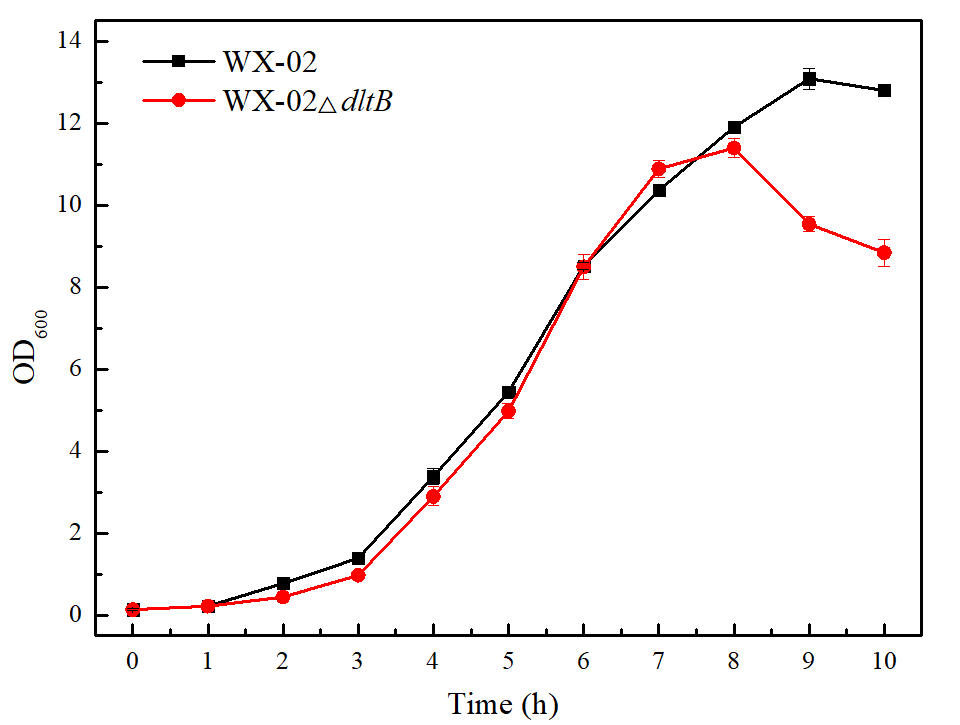


**Fig. S1**

**Fig. S2 The transcriptional levels of *dlt* gene in single gene deletion strains.**

**
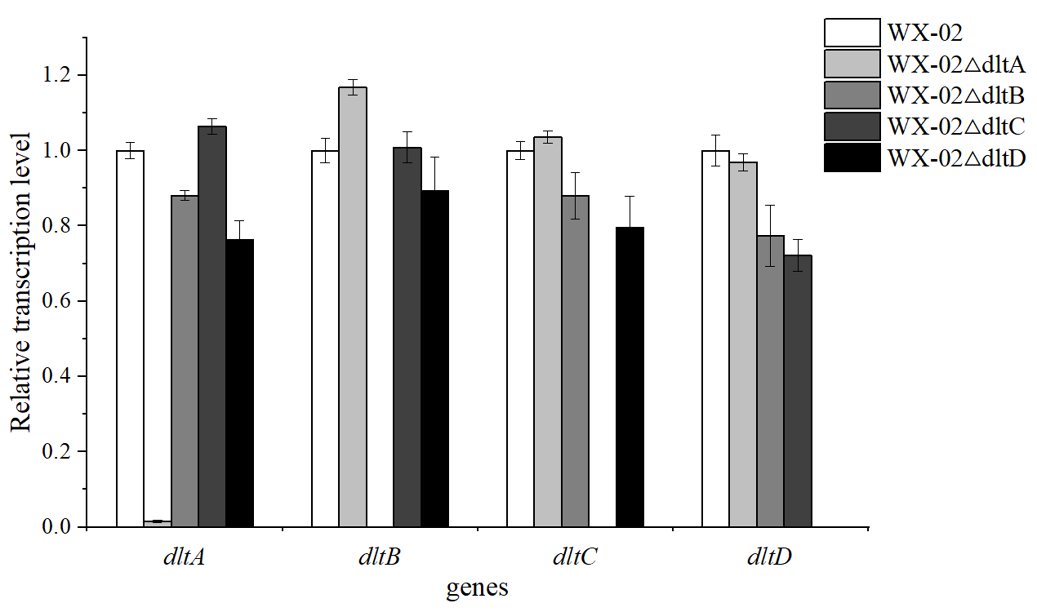
**

**Fig. S2**

**Fig. S3 Effects of overexpression of *dlt* operon on the synthesis of lichenysin, bacitracin and pulcherrimin.**

**
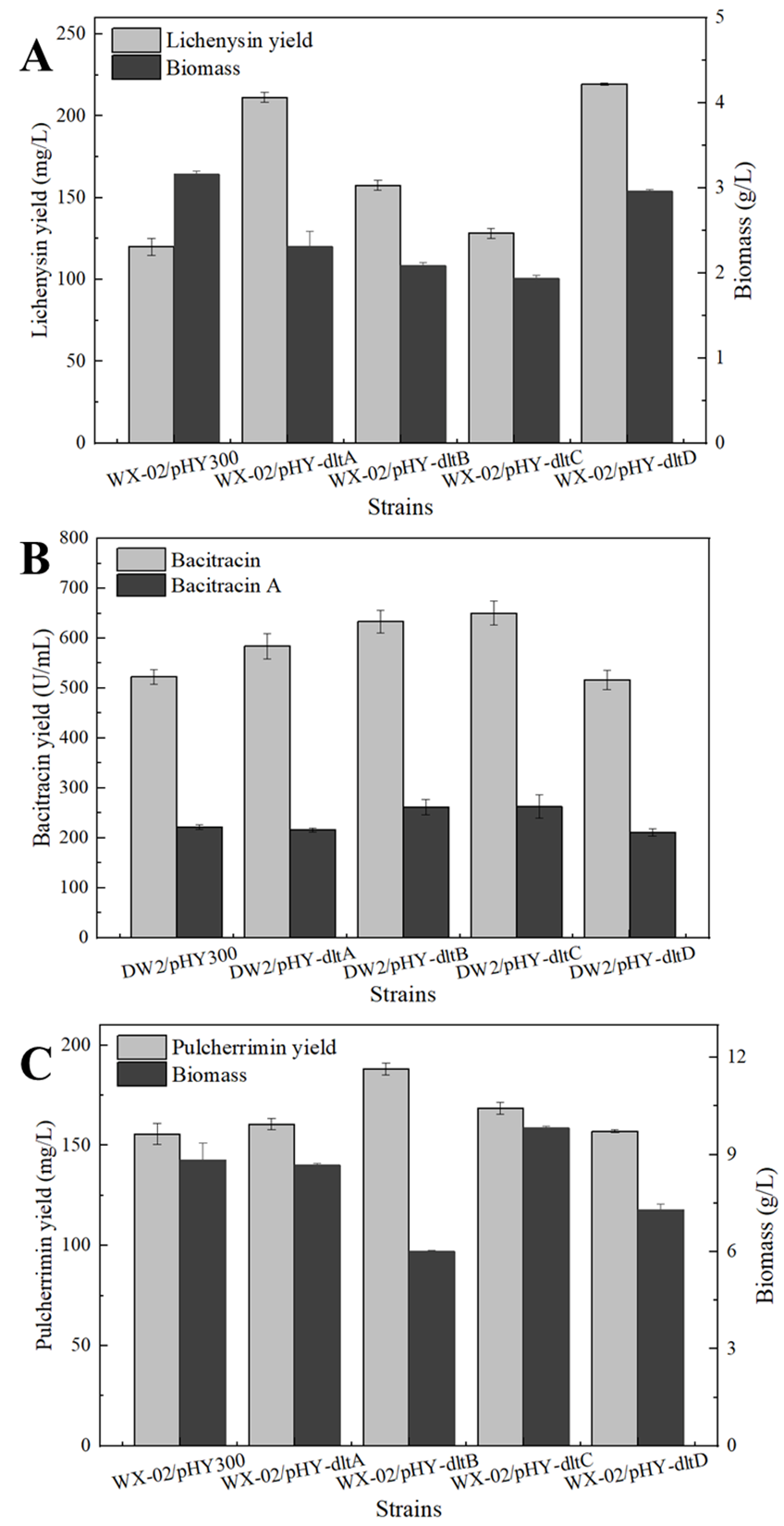
**

**Fig. S3**
